# Ionovoltaic electricity generation over graphene-nanoplatelets: protein-nanofibril hybrid materials†‡

**DOI:** 10.1039/d2na00388k

**Published:** 2023-01-10

**Authors:** Lei Wang, Lianlian Liu, Niclas Solin

**Affiliations:** a Division of Electronic and Photonic Materials, Biomolecular and Organic Electronics Unit, Department of Physics, Chemistry, and Biology, Linköping University Linköping 5 81 83 Sweden niclas.solin@liu.se

## Abstract

Continuous harvesting of electricity from the ambient environment has attracted great attention as a facile approach to green and sustainable energy. Natural water evaporation-driven electricity generators with active materials from economical and environment-friendly sources are highly sought after. Herein, we present devices made from a combination of protein nanofibrils (PNFs) and low-cost graphene nanoplatelets (GNPs) that can be employed for electricity generation, simply by partly inserting the device into evaporating standing water. The origin of the electricity generation can be explained by the ionovoltaic effect where ionic motion, driven by evaporating water, leads to movement of charge carriers in the electrically conductive GNP-phase. Moreover, the device performance can be improved by adding a small amount of salt to the active layer. A device, composed of GNP:PNF:AlCl_3_, produces a sustained voltage of about 0.48 V, and a current of 89 nA. Furthermore, the device can tolerate saline water, with only a modest decrease of voltage, which provides potential for harvesting electricity from both evaporating saline water and fresh water.

## Abstract 2

Devices with an active layer made from protein nanofibrils, graphite nanoplatelets and salts can harvest electricity from evaporating water.

## Graphical abstract

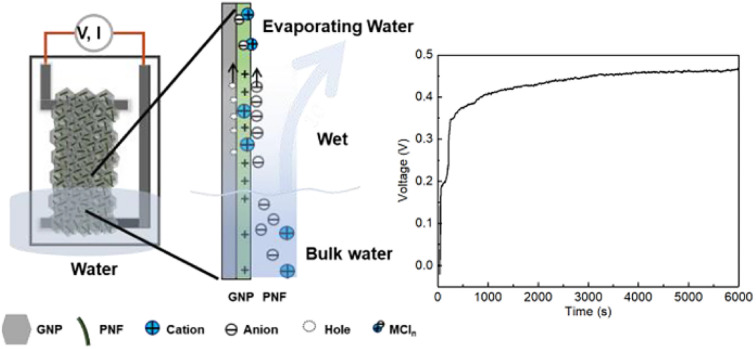

## Introduction

It is estimated that water covers about 71% of the Earth's surface; water is moreover present in both liquid and gaseous form under normal conditions meaning that water is circulated by the hydrological cycle. As a type of renewable energy source, devices that can harvest electricity from water in the ambient environment are highly sought after.^1,3–7^ Moreover, as saline water makes up 97% of the Earth's water it is desirable to develop electricity generators that can operate with saline water. Water is highly polar liquid with a unique capacity to stabilize charged species (such as salts, acids, and bases). The separation of solvated cations and anions will generate electric fields, and if a macroscopic asymmetric ion distribution is achieved, this will result in an electromotive force. This can be employed to generate a voltage between two electrodes in a device, which will drive a current when the device is connected to an external circuit.

The processes that can be employed to generate asymmetric ion distributions are related to achieving different mobilities of anions and cations. One process is based on the Soret effect,^9^ where a temperature gradient will lead to an enrichment of the more mobile ion in the cold area. Another approach is to employ systems with non-mobile charges (for example by having the cation or anion attached to a stationary phase); movement of water may then lead to selective transport of the mobile counterion, giving rise to an electric field.^12^ This phenomenon was reported already in 1859 by Quincke, who found that an electrical potential is generated between the ends of a tube when water is pumped through narrow pores (made from a variety of materials such as glass, silk, and linen).^13,14^ Moreover, the presence of stationary charges may influence the relative mobility of cations and anions, that may further promote the generation of an asymmetric ion distribution. Regardless of their origin, if such processes can be coupled with charge transport in a conductive phase, the result is a device that may convert water movement into an electric current. In such systems ionic movement in the aqueous phase provides the mechanism for formation of an electromotive force, that can in turn induce movement of electrons and holes in the conductive phase. As ionic movement in the aqueous phase combined with interfacial ion–charge carrier interactions lead to movement of charge carriers in the conductive phase (providing a voltage) this type of process may appropriately be labeled as an ionovoltaic process.^8,12,15–17^ It should be noted that similar processes have been labeled as hydrovoltaics in many recent publications, even though ionic species often are involved in the operating mechanisms. Regardless of the choosen label, this approach has recently received much interest; for example, by employing carbonaceous materials such as carbon nanotubes and graphene as electrically conductive phase. When incorporated into devices these carbon nanostructures generate electricity when interacting with flowing, waving, and dropping water.^4,6,18,19^ Many studies have employed microfluidic devices where water is made to flow over graphene or carbon nanotubes.^6,20–23^ It has also been shown that devices can generate electricity induced by movement of charged droplets on a graphene sheet, or conversely by movement of a graphene sheet in water.^24,25^ An intriguing recent finding is that ambient evaporation of water from nanostructured carbon materials can generate a continuous electric current.^1,3,4^ Under these conditions water evaporation is driven by the ambient heat, meaning that such devices indirectly convert the ambient thermal energy into electricity and may accordingly potentially function as off-grid autonomous power sources for low power electronic devices. In 2017 it was reported that ambient water evaporation from centimeter sized carbon black sheets (connected to electrodes) could produce an electric voltage of up to 1 V and a current of up to 100 nA.^1^ The mechanism is believed to be related to rise of water into cavities in the carbon black film. Carbon black contains ionizable functional groups, and upon capillary rise of water, there will be a net flux of ions (of opposite charge to the functional groups attached to carbon black) generating a voltage. When connected to an external circuit this allows a continuous electric current to flow. It has also been shown that surface modification of carbon black with polyelectrolytes can lead to increased voltages, and the sign of the voltage can be modified by employing polyelectrolytes with either positive or negative stationary charges.^26^ In addition, nanostructured carbon,^27^ printed carbon black films,^2^ conductive polymers modified carbon black,^28–30^ and graphene oxide sponges^31^ have been demonstrated to generate electricity. However, a drawback with most of these devices is that they require deionized water for optimum function. The reported carbon-based evaporation devices have had active layers made from carbon black/graphene oxide materials with rather high resistivity,^1,8^ which might limit the possibilities to tune the properties of the materials by forming hybrids with insulating components. It is hence interesting develop graphene-based materials (with a relatively high electrical conductivity) as it gives more possibilities for development of hybrid materials where the conductive carbon phases are combined with electronically insulating materials providing stationary charges.

We have recently developed methodology based on mechanochemistry for exfoliation of graphite into graphene nanoplatelets (GNPs) in the presence of protein nanofibrils (PNFs), resulting in aqueous dispersions of GNP:protein hybrids.^32^ These GNPs show electrical conductivity up to 110 S m^−1^ (which is relatively high compared with graphene oxides and reduced graphene oxide^33^) and the aqueous GNP dispersion can easily be processed into thin films. We have earlier reported that thin (dry) films of hybrids between GNPs and protein nanofibrils (PNFs) can generate a voltage when exposed to a temperature gradient, but the presence of electrically insulating PNFs proved detrimental to performance.^32^ However, from the standpoint of generating electricity from evaporating water the presence of proteins in the GPN:PNF hybrids have beneficial aspects, as proteins have ionizable groups such as carboxyl groups and amino groups, that may act as stationary charges. In the present study PNFs formed from Hen egg white lysozyme (HEWL) were employed. HEWL is a readily available low-cost protein (used by the cheese industry and brewers, with E-number E1105) and has a high isoelectric point (pI = 10.7). Accordingly, HEWL can be expected to provide positively stationary charges and mobile negatively charged counter ions in neutral water.^34^ HEWL can readily be converted into HEWL PNFs simply by heating in acidic water.^35,36^ Such PNFs typically have diameters of 5-10 nm and lengths in the micrometer-range.^37^

In this study, we have formed hybrid materials between GNPs and HEWL PNFs, in order to fabricate devices with a GNP:PNF active layer that can harvest electricity from standing water at ambient conditions. Salts (NaCl, CaCl_2_, AlCl_3_) may be incorporated into the active layer to tune the electrical properties of the active layer and the hygroscopicity of the device surface. The properties of the GNP:PNF hybrids may be further tuned by adding extra PNFs thus adjusting the relative ratio of GNPs and PNFs. Among the employed salts, sodium chloride is abundant and mainly responsible for the salinity of seawater,^38^ calcium chloride, the main ingredient of desiccants, absorbs moisture from surrounding air,^39^ and aluminum chloride, has a very pronounced affinity for water. When dissolved in water, the different salts dissociate into chloride ions and the corresponding cations. However, it should be noted that Al^3+^ in water has a complex chemistry and a wide range of hydrated species can form.^40,41^ By incorporating salts into the GNP:PNF hybrids the device performance is improved, and devices with an active layer consisting of GNP:PNF:AlCl_3_ displayed a voltage of 0.46 V and a current of 89 nA. The devices can also operate in saline water but with a reduced voltage.

## Results and discussion

**Fig. 1:**
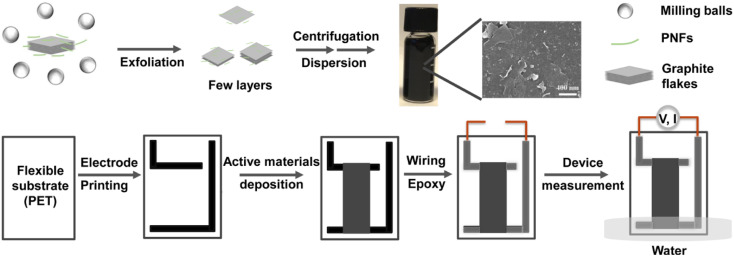
Schematic illustration of preparation of GNP-based materials and devices.

A graphical overview of the preparative method to obtain GNP dispersions, as well as the fabrication method to obtain GNP-based devices for generation of electricity from evaporating standing water is presented in Fig. 1. (Note that the preparative method and basic characterization of the GNP:PNF hybrids have been reported in the previously mentioned study of the thermoelectric properties of the materials.^32^) In brief, GNPs are prepared by ball milling of an aqueous dispersion of HEWL PNFs and graphite, resulting in exfoliation of graphite and formation of an aqueous dispersion of GNPs, where PNFs helps to disperse GNPs by electrostatic stabilization. Large graphite particles are then removed by centrifugation at low speed. Excess PNFs are then removed by centrifugation at high speed, resulting in a GNP-enriched pellet. Redispersion of the pellet in water results in a black colored aqueous GNP-ink (10 mg mL^−1^). Note that, in common with other studies, not all the dispersing agent (*i.e.*, PNFs) will be removed during this procedure, meaning that the GNP-ink contains residual PNF materials, see below for further discussion. For device fabrication, a PET substrate is patterned with L-shaped conductive carbon electrodes (Fig. S1, (ESI‡)). A 3 × 1 cm^2^ mold is then created by applying scotch tape, and the aqueous solution of selected active materials (an appropriate mixture of GNP-ink, additional PNFs and salts) is deposited onto the substrate. The device is heated at 60 °C for about 30 minutes, resulting in a dry 3 × 1 cm^2^ film. After removal of the scotch tape mold the electrodes are equipped with wiring that is sealed by epoxy glue, in order to avoid contact with water. The device is then partly inserted into water for investigation of electricity generation from evaporating standing water. The device configuration is designed to avoid insertion into water of the contact points between the carbon electrodes and the wiring.

### Characterization of GNP inks

**Fig. 2:**
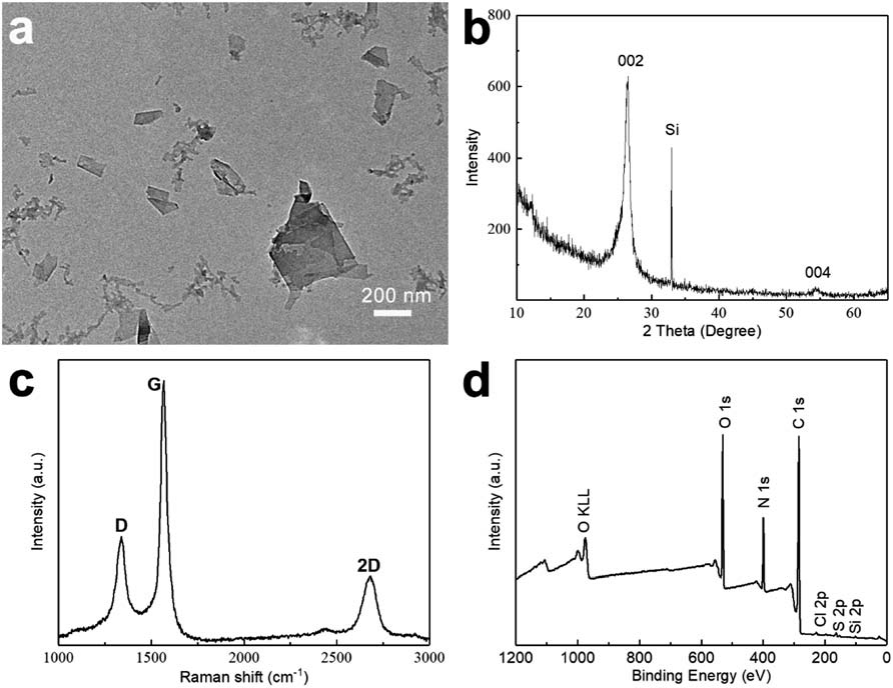
GNP-ink characterization: TEM image (a), XRD pattern (b), Raman spectrum (c), and XPS survey spectrum (Si element comes from the Si substrate) (d).

A typical TEM image obtained from a GNP-ink sample, deposited onto a TEM-grid by dip-coating, is shown in Fig. 2a. A number of flake-like structures can be observed, with a morphology typical of graphene-like sheets. Fig. 2b shows the XRD pattern obtained from a sample of dried GNPs on a Si substrate, exhibiting a peak at 2 theta = 26.6°, which indicates the basal inflection (002 plane) in graphite crystals (originating from the interlayer distance between graphene sheets) and a small peak at 2 theta = 54.7°, which indicates a long-range order of graphene layers.^42^ Raman spectroscopy was employed in order to evaluate the presence of structural defects in the GNPs. The intensity ratio of the D band and the G band (*I*_D_/*I*_G_) is usually used to extract the concentration of defects, and thus indicate the quality of carbon materials.^43,44^ As shown in Fig. 2c, the *I*_D_/*I*_G_ ratio is 0.41, demonstrating that the preparative method gives GNPs of good quality with few defects.^45–47^ The 2D band is located at about 2678 cm^−1^, which is below 2720 cm^−1^, indicating that the GNPs consist of less than 10 layers.^48^ In order to investigate the elemental composition of the GNP-ink, the samples were analyzed by XPS. The XPS survey spectrum of a GNP-ink sample shows the presence of C, N O, S and Cl elements. For reference XPS survey spectra of graphite and HEWL PNFs are provided in Fig. S2.‡ The XPS spectrum of graphite shows the presence of C and O, and HEWL PNF spectrum contains peaks corresponding to C, O, N, S and Cl elements, which demonstrates that the GNP-ink contains HEWL PNF residues. The GNP-ink has a zeta potential of +45 mV, which is near to the zeta potential of HEWL PNFs in water (+47 mV), indicating the surface net charge of GNPs is dominated by the HEWL PNF residues (Fig. S3‡).

### Characterization of GNP-based devices

The GNP-ink can readily be processed into thin films (the active layer of a device) by casting in a mold, followed by evaporation of water. Devices can readily be prepared in the same manner, simply by casting the film on a substrate prepatterned with two electrodes. As described above, the original GNP-ink contains residual PNFs, originating from PNFs functioning as dispersing agents (during milling of graphite in the presence of PNFs). During the milling step, where graphite is exfoliated in the presence of PNFs, the PNFs are cut into short fibrils and have a typical length of about 200 nm;^32^ however, the PNFs added to the original GNP-ink have not been milled and are hence much longer, with typical lengths of several micrometers (Fig. S4 in ESI‡). By mixing the GNP-ink with PNFs and/or salts during the casting process the composition of the active layer can be modified. An active layer with a relatively large amount PNFs (thus giving a low GNP : PNF ratio) will contain a large amount of charged functional groups. This provides one parameter that can be varied in order to tune the performance of the device. In addition, the device performance can be tuned by incorporation of salts in the active layer. In a preliminary study we found that a GNP : PNF mass ratio of 5 : 1 gave the best performance when adding salts (Fig. S5‡). The active layer was prepared by adding a PNF dispersion into the original GNP-ink, and salts (NaCl, CaCl_2_ and AlCl_3_) were added as aqueous solutions (prepared by dissolving the different salts in water). Note that in the case of CaCl_2_ and especially AlCl_3_ the cation is likely present in a hydrated form, also after drying.

**Fig. 3:**
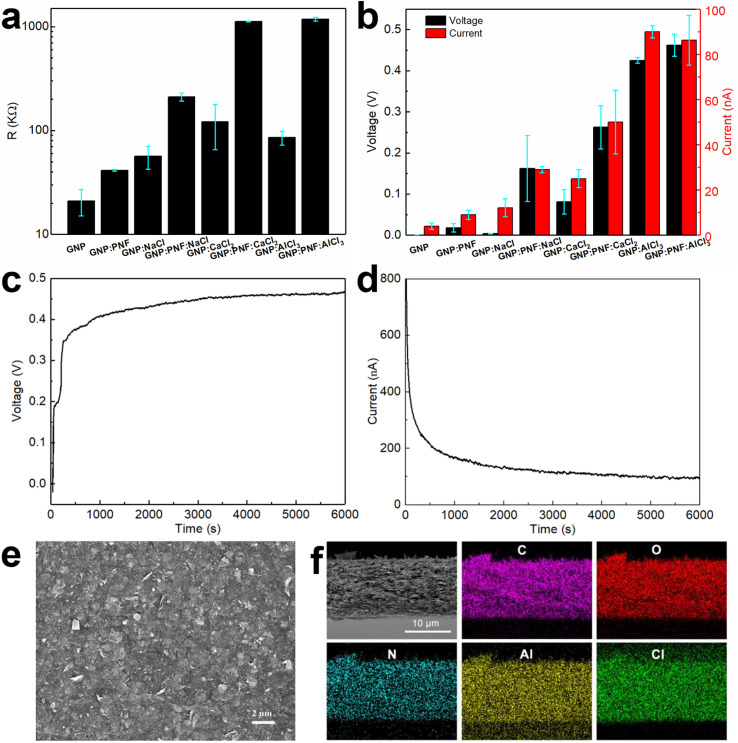
Resistance *vs.* GNP-based devices (a); *V*_oc_ and *I*_sc_*vs.* GNP-based devices (b) (note that the *I*_sc_ is the steady current). *V*_oc_ (c) and *I*_sc_ (d) of GNP:PNF:AlCl_3_ device as a function of time operating in water reservoir in ambient condition. SEM image of surfaces (e) and cross section of a GNP:PNF:AlCl_3_ film and carbon, oxygen, nitrogen, aluminum, and chlorine mapping of the same position (f).

The devices are named generally as GNP:PNF:salt and specifically as GNP:PNF:NaCl, GNP:PNF:CaCl_2_ and GNP:PNF:AlCl_3_, respectively. We also investigated the effect of addition of salt to the original GNP-inks, by the addition of the same salts into the original GNP-ink (without additional PNFs). These devices are labeled generally as GNP:salt, and specifically as GNP:NaCl, GNP:CaCl_2_ and GNP:AlCl_3_, respectively. The control, consisting of the GNP-ink (without added PNFs or salt) is labeled as GNP. It should be noted that, as mentioned above, the active layers in the GNP and GNP:salt devices will still contain a small amount of short PNFs. The resistance was investigated for the different types of devices, in their dried state, and the results are shown in Fig. 3a. The GNP shows a resistance of 22 kΩ, and the resistivity of GNP:PNF increases due to the electrically insulating character of PNFs. When comparing the devices with different salts in the active layer, GNP:PNF:AlCl_3_ and GNP:PNF:CaCl_2_ devices are quite similar with a resistance of 1200 kΩ, and the resistance of GNP:PNF:NaCl is 200 kΩ, which might be due to the finer morphology of the GNP sheets in the active layer compared to the other two cases (Fig. S8a, c and e‡). The devices with active layers consisting of GNP:salt all have quite similar resistances in the range of 60 to 100 kΩ. The resistances of all the different GNP:salt active layers are lower than the corresponding resistances for the GNP:PNF:salt active layers due to the presence of electronically insulating PNFs. When partly inserted into a water reservoir the devices display a voltage and generate a current if the circuit is closed (Fig. 3b–d). The voltage and current characteristics of the different devices were characterized by measuring the open circuit voltage (*V*_oc_), and the short circuit current (*I*_sc_), and the results are shown in Fig. 3b. When the original GNP-ink is employed as active layer (GNP) a *V*_oc_ of 0.5 mV and *I*_sc_ of 3 nA is obtained. When PNFs are introduced in the active layer (GNP:PNF), the *V*_oc_ and *I*_sc_ are 0.04 V and of 10 nA. Addition of PNFs accordingly increases *V*_oc_ by roughly an order of magnitude. For devices prepared from GNP:PNF:salts and GNP:salts, the *V*_oc_ and *I*_sc_ of GNP:PNF:NaCl are 0.162 V and 29 nA, while the *V*_oc_ and *I*_sc_ of GNP:NaCl are 3.5 mV and 12 nA. The *V*_oc_ and *I*_sc_ of GNP:PNF:CaCl_2_ are 0.263 V and 50 nA, while the *V*_oc_ and *I*_sc_ of GNP:CaCl_2_ are 0.081 V and 25 nA. For GNP:AlCl_3_, the *V*_oc_ is 0.43 V. The *I*_sc_ are quite similar for GNP:PNF:AlCl_3_ and GNP:AlCl_3_ (both about 90 nA). The GNP:PNF:AlCl_3_ achieves a highest *V*_oc_ of 0.46 V (Fig. 3c), among all the GNP-based devices, and the *V*_oc_ values have a clear trend for the same molal of added salt, with AlCl_3_ > CaCl_2_ > NaCl. From these results it is readily apparent that addition of PNFs and salt into the active layer of the device is beneficial for device performance. Moreover, the results show that the electricity generation in devices with active layers consisting of GNP:salt and GNP:PNF:salt depends on the type of salt employed. In the case of NaCl and CaCl_2_ the addition of PNFs results in a significant improvement of both *I*_sc_ and *V*_oc_. On the other hand, in the case of AlCl_3_ the addition of PNF is less critical, as addition of PNFs leads only to a slight increase of *V*_oc_ and a slight decrease of *I*_sc_. The *I*_sc_*versus* time data was collected after the initial measurement of *V*_oc_ for 6000 s. Interestingly, for all GNP-based devices (as shown in Fig. 3d, S6 and S7‡) *I*_sc_ starts from a high level (several hundreds of nA), followed by a dramatic decrease lasting for about 500 s. There is then a period of slow change before finally a steady level is reached. As a control experiment, the *I*_sc_ was collected just after the GNP:PNF:AlCl_3_ device had been inserted into the water reservoir (Fig. S8‡). In this case there is an initial rapid increase in current, followed by a rapid decrease, which is a similar result to those obtained when *I*_sc_ is measured after the *V*_oc_.

The microscopic structure of the active layer in the GNP-based devices was investigated by SEM (Fig. 3e, f and S9–S11‡). The surface morphology of GNP:PNF:AlCl_3_ is shown in Fig. 3e, and reveals the presence of sheet structures from the GNPs assembled on the substrate. SEM images of the surface region of the active layer formed from different combinations of GNP, PNF, and salts are shown in Fig. S5 and S9.‡ All the images show similar surface texture, which is similar to the surface of GNP:PNF:AlCl_3_ (Fig. 3e). As shown in Fig. 3f, S10 and S11‡ the thicknesses of the active layer of the different GNP:salt, GNP:PNF and GNP:PNF:salt devices vary: 2.7 μm for GNP:NaCl and 3 μm for GNP:PNF:NaCl, which is quite similar to the thickness of GNP:PNF (2.8 μm); 4.7 μm for GNP:CaCl_2_ and 4.3 μm for GNP:PNF:CaCl_2_. The thickness is 3.3 μm for GNP:AlCl_3_ and 12 μm for GNP:PNF:AlCl_3_. The variations in thicknesses indicate that the added salts influence the film morphology, which is likely due to the ability of salts to coordinate to PNFs^49–51^ as well as the ability of Al^3+^ to form hydrates. To determine the homogeneity of the active layer components, the devices with the best performance were selected. EDX mapping images of GNP:PNF:AlCl_3_ (Fig. 3f) show that the elements C, O, N, Al, and Cl are uniformly distributed in the whole film, indicating that GNP, PNFs and Al^3+^ are homogeneously mixed in the active layer. A similar homogeneous mixture is also observed for GNP : AlCl_3_ as shown in EDX mapping images (Fig. S11‡). As pointed out earlier, AlCl_3_ is likely present in a hydrated form, with water and suitable amino acid moieties coordinating to Al^3+^.

### Power generation evaluation

**Fig. 4:**
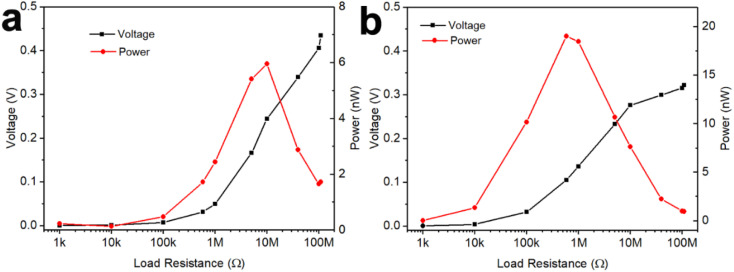
Power and voltage of a GNP:PNF:AlCl_3_ (a) and a GNP:AlCl_3_ (b) device on an alterable load resistor.

The GNP:PNF:AlCl_3_ device was investigated as a power source to estimate the maximum output power, as shown in Fig. 4a. When the load resistance is varied from 1 kΩ to 110 MΩ, the output voltage increases from 0 to 0.42 V. The output power was calculated by the equation *P* = *V*^2^/*R*. The output power of GNP:PNF:AlCl_3_ increases with a load resistance, until the resistance is 10 MΩ, and then decreases as the load resistance is further increased. The maximum output power is 6 nW. The loading mass and volume of GNP:PNF:AlCl_3_ active layer were 2.2 mg and 3.6 × 10^−3^ cm^3^, respectively. The maximum output specific power and power density was calculated to be 2.73 nW mg^−1^ and 1.67 μW cm^−3^, respectively. The GNP:AlCl_3_ device shows a relatively high maximum output power of 19 nW with an external resistance of 500 kΩ (Fig. 4b), and the maximum output specific power and power density was calculated to be 10 nW mg^−1^ and 19 μW cm^−3^, respectively, with a loading mass of 1.9 mg and volume of 1 × 10^−3^ cm^3^. Compared to GNP:PNF:AlCl_3_, the GNP:AlCl_3_ has a higher electrical conductivity in the dry state as shown in Fig. 3a. The GNP:AlCl_3_ has a higher electronic conductivity and thus more electrons/holes will be transported into the external circuit which may explain the higher maximum output power observed for the GNP:AlCl_3_ system. The absolute output power may be lower than most of the published results (as listed in Table 1). However, when considering of the low loading mass and volume, the output specific power and power density of the GNP:PNF:AlCl_3_ and GNP:AlCl_3_ are comparable and superior to most of the listed electricity generators, which gives a potential for further development of the GNP:PNF:AlCl_3_ and GNP:AlCl_3_ devices.

**Table 1:** Summary of related performance parameters of the water evaporation electricity generating devices

Active materials	*V*_oc_ (V)	*I*_sc_ (nA)	Maximum absolute output power (nW)	Specific power (nW mg^−1^)	Power density (μW cm^−3^)	References
Nanostructured carbon	Up to 1	100				1
Toluene soot	Up to 1	600	172	0.172	8.1	2
ZnO	0.38	6	2			8
Functionalized CNT	0.385	6800	27 600	4600	27 × 10^3^	10
Metal organic framework	1.63	∼500	150	3	6.5	11
GNP:PNF:AlCl_3_	0.48	89	6	2.73	1.67	This work
GNP:AlCl_3_	0.44	90	19	10	19	

### Mechanistic discussion

**Fig. 5:**
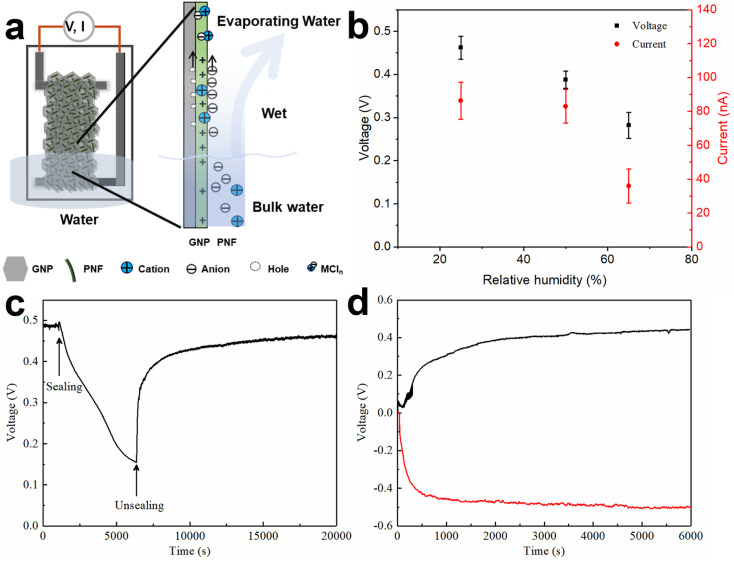
Schematic image of the water evaporation induced electricity generation in device of GNP:PNF:AlCl_3_ as an example (a), *V*_oc_ and *I*_sc_ as a function of time for GNP:PNF:AlCl_3_ operating in different relative humidity (RH) (b), *V*_oc_ as a function of time for GNP:PNF:AlCl_3_ operating in water reservoir in open and sealing conditions (c); switch effect on GNP:PNF:AlCl_3_: the *V*_oc_ as a function of time for before (black curve) and after upside down (red curve) conditions (d).

A possible explanation of the generation of electricity during water evaporation is related to the ionovoltaic effect,^12,15,16^ where ionic movement is coupled with movement of charge carriers in the conductive (GNP) phase of the device, as shown in Fig. 5a and S12.‡ When the device is inserted into the water reservoir, water molecules will diffuse into the active layer. The active layer is a hybrid between GNPs and PNFs, and the PNF concentration can be tuned by addition of extra PNFs. PNFs have diameters of about 10 nanometers and lengths in the micrometer range. The rope-like shape PNFs may facilitate formation of elongated pore-like structures that may enable water transport within GNP:PNF hybrids.^52^ Alternatively, water molecules may undergo diffusion within the PNF matrix.^53,54^ HEWL PNFs have a zeta potential of +47 mV (Fig. S3b‡), which indicates a strong net positive charge; the PNFs will hence provide stationary positive charges and mobile counter ions. As the PNFs were prepared in 25 mM HCl the stationary charges will originate from protonated basic amino acid residues with mobile Cl^−^ as counter ions. If non-protonated basic functional groups remain, these may deprotonate water, again leading to a stationary ammonium group but now with HO^−^ as counterion. As shown in Fig. S5b,‡*V*_oc_ has a clear dependence on the amount of PNF. For the GNP:PNF:salt and GNP:salt devices, before inserting into water, the active layer contains added salt (NaCl, CaCl_2_, or AlCl_3_) and PNF with positive charges and associated counter ions. When the device is partly inserted into water the salt will dissolve upon contact with water and form cations and Cl^−^. In this case both the cations and anions (Cl^−^) are mobile. The active layer above the stationary water level (wet part in Fig. 5a right, and Fig. S12‡) will be infiltrated with water and hence become wet. This means that the salt will dissociate into cations and anions. Due to the high concentration of stationary positive charges provided from PNFs the active layer will display a high counterion permselectivity. The cations (and especially the multivalent cations) will experience strong electrostatic repulsion (from the PNF stationary positive charges) that will hamper their movement. Evaporation of water will lead to a net flow of water towards the top part of the device, which in turn will lead to a concomitant transport of mobile Cl^−^ ions. The movement of chloride ions is coupled with the movement of holes in the GNP phase (through a coulombic drag mechanism^18^); holes in the GNP phase can accordingly be accumulated towards the top of the active layer leading to an electrical imbalance that will manifest itself in the form of a voltage at the electrodes. Constant water circulation enabled by water evaporation could then provide a stationary voltage. It should be noted that as the cations in the employed salts (NaCl, CaCl_2_, or AlCl_3_) have different valency, for the same molar ratio the trivalent Al^3+^ supplies 3 times of Cl^−^, divalent Ca^2+^ supplies twice of Cl^−^ compared to the amount of Cl^−^ the Na^+^ supplies. Moreover, the mobility of cations and anions are different. The hydrodynamic radii of hydrated Al^3+^, Ca^2+^ and Na^+^ are 1.510 Å, 1.548 Å and 1.840 Å respectively, which are larger than the hydrodynamic radius of Cl^−^ (1.245 Å).^55^ If we assume normal drag behavior this fact alone would lead to enrichment of Cl^−^ along the direction of water movement.

A complementary mechanism for voltage generation is leakage of ions from the part of the device inserted into the bulk water: when a GNP:PNF device is in operation, the lower part of the active layer is inserted directly in water, meaning that the PNFs and their counter ions (Cl^−^) get in direct contact with water. The mobile Cl^−^ will gain entropy when diffusing into the surrounding water, while the positively charged PNFs are essentially immobilized in the active layer; a process that will also promote formation of a net electric field (voltage).

In addition to movement of ions induced by water transport, another possibility is movement of ions due to temperature differences (the Soret effect). When exposing a GNP:PNF:AlCl_3_ to 99% relative humidity atmosphere, and two ends of the device exposed to temperature difference of 20 °C, which is much larger than the temperature difference resulting from evaporation of water at ambient condition,^56^ the voltage was about 0.08 V (Fig. S13‡), which is only 17% of the voltage obtained from this type of device when inserted in water (0.46 V). Accordingly, the thermoelectric effect makes a negligible contribution during device operation.

An additional important effect relevant for devices with active layers incorporating AlCl_3_ and CaCl_2_ are the hygroscopicity of these salts.^39^ AlCl_3_ and CaCl_2_ are both deliquescent and their presence in the active layer allows an uptake of a greater amount of water in the film as shown in Fig. S14 in ESI,‡ where the mass changes are plotted when exposing films to 65% relative humidity as a function of time. Among all the GNP:PNF:salt films, the mass gain from moist air is as follows: GNP:PNF:AlCl_3_ (31 mg cm^−2^) > GNP:PNF:CaCl_2_ (26 mg cm^−2^) > GNP:PNF:NaCl (16 mg cm^−2^). In addition, the GNP:PNF:AlCl_3_ film takes up more water than GNP:AlCl_3_ films (20 mg cm^−2^), which indicate that the presence of PNFs allows for more water to be absorbed.

To better understand the electricity generation process, the influence of the external conditions was further investigated. When exposing a GNP:PNF:AlCl_3_ device to a relative humidity of 96%, without inserting the film into water (Fig. S15‡), the *V*_oc_ and *I*_sc_ were 15 mV and 2 nA, respectively. We also tested the effect of varying the relative humidity of the ambient air for a GNP:PNF:AlCl_3_ was inserted into water. As shown in Fig. 5b, the voltage and current were 0.46 V and 90 nA, respectively, when the relative humidity was 25%. When the relative humidity increased to 50% and 65%, the voltage decreased to 0.38 V and 0.3 V, and the *I*_sc_ were 95 nA and 50 nA, respectively. Accordingly, the performance of GNP:PNF:AlCl_3_ shows a strong humidity dependance. At lower relative humidity, water will evaporate more rapidly from the film, resulting in more water transport in the film, inducing a higher voltage.

Fig. 5c shows the voltage changes of a GNP:PNF:AlCl_3_ device operated under open and closed conditions (where the beaker was sealed with a plastic film). The *V*_oc_ is 0.49 V under open condition. When the device was sealed, the *V*_oc_ gradually decreased to 0.15 V (after 5000 s), which is related to the water vapor gradually reaching saturation. When the cover is removed, the voltage rapidly recovered. These results demonstrate that water evaporation is the key process for voltage generation in the GNP:PNF:AlCl_3_ device. The result from a switch polarity test is shown in Fig. 5d. When a GNP:PNF:AlCl_3_ device is partly inserted in water, a *V*_oc_ of 0.44 V was observed (black curve). After being operated for 6000 s, the same device was dried and turned upside down with the previous top end now being inserted into water (without changing any electrical connections). The voltage (red curve) reached a similar value (0.48 V) but with a opposite sign. The result shows that both sides of the device function in a similar manner, further indicating that water evaporation is the key process for the voltage generation.

### Effect of salt in the water reservoir

**Fig. 6:**
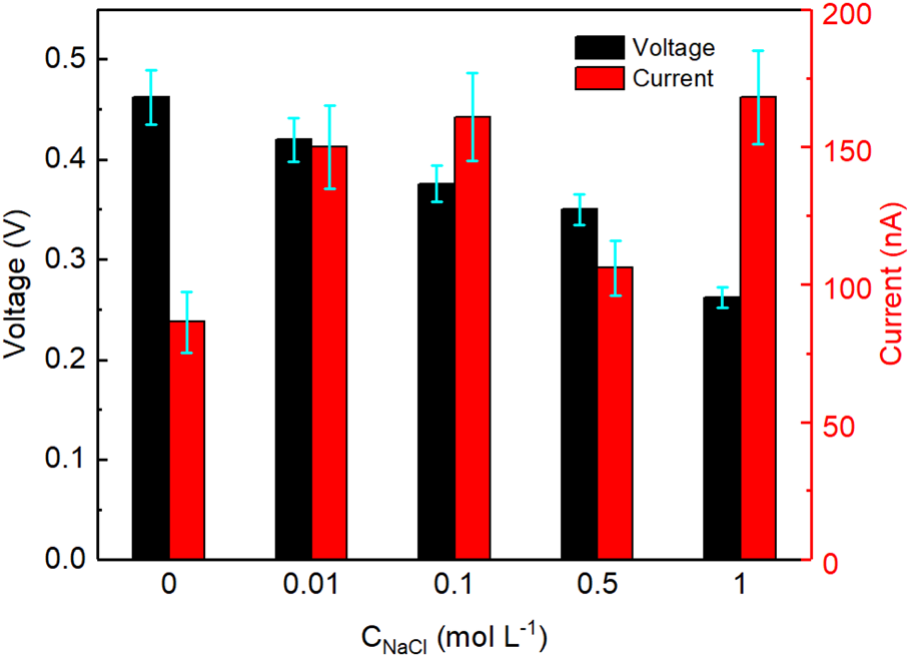
*V*_oc_ and *I*_sc_ as a function of time for GNP:PNF:AlCl_3_ devices operating in different concentrations of NaCl solution.

The devices described above were all tested by partly inserting the device in a reservoir containing DI water. To study the effect of saline water on the electricity generation of GNP:PNF:AlCl_3_ devices, salty water (with different concentrations of NaCl) were employed instead of DI water, and the results are shown in Fig. 6. As a reminder, when a GNP:PNF:AlCl_3_ is inserted in reservoir containing DI water, the observed voltage was 0.46 V. In the case of salty water, the voltage was observed to be 0.42, 0.38, 0.35 and 0.25 V, in 0.01, 0.1, 0.5 and 1 mol L^−1^ of NaCl, respectively. The *I*_sc_ was observed to be 150, 160, 120 and 170 nA in 0.01, 0.1, 0.5 and 1 mol L^−1^ of NaCl, respectively, which is an enhancement compared to the device operating in DI water (89 nA). The performance of devices is accordingly significantly influenced by the salinity of standing water, with the key results being that the devices still show a decent performance, with a drop of *V*_oc_ coupled with an increase in *I*_sc_ as a function of increased salt concentration. When a GNP:PNF:AlCl_3_ device is inserted in the reservoir containing salt, the NaCl solution will influence the electrical double layer at the solid/liquid interface. The active layer of the GNP:PNF:AlCl_3_ device contain positively charged PNFs. According to the Debye–Hückel theory,^57^ the Debye length decreases as the ionic concentration increases, which may lead to a decrease of the selectivity of ionic transport and hence a reduced *V*_oc_. In addition, the presence of Cl^−^ ions in the reservoir will lead to a higher concentration of ions in the active layer, which may lead to an increase of the *I*_sc_. The above results demonstrate that GNP:PNF:AlCl_3_ devices function efficiently in the presence of moderate salt concentrations, and may accordingly be employed also in natural waters such as lakes and streams.

## Conclusions

We conclude that GNP-based devices are interesting candidates as electricity generators in standing water, with water evaporation providing a driving force for continuous generation of electricity. The active materials are prepared from naturally abundant graphite and biobased polymer protein nanofibrils. By incorporating salt into the GNP and GNP:PNF active layer, the device performance is highly improved. The device made from GNP:PNF:AlCl_3_ can achieve a *V*_oc_ of 0.46 V and a *I*_sc_ of 89 nA and a maximum absolute output power of 6 nW. The device made from GNP:AlCl_3_ can achieve a *V*_oc_ of 0.44 V and an *I*_sc_ of 90 nA and a maximum absolute output power of 19 nW. With a maximum output specific power of 10 nW mg^−1^ for GNP:AlCl_3_ and 2.73 nW mg^−1^ for GNP:PNF:AlCl_3_, which is superior to most of other carbon or metal oxide/MOF based devices (Table 1). Importantly, the devices show only a small decrease in performance when operating in saline water. Our results demonstrate that protein nanofibrils may play an important role in the development of devices that can harvest electricity from the ambient environment.

## Experimental

### Materials

Graphite flakes (product number 332461) were purchased from Sigma-Aldrich. Lysozyme from hen egg white were bought from Caglificio Clerici, Cadorago, Italy. All chemicals were used as received without further purification. Ultrapure water (18.2 MΩ cm) was obtained from a Milli-Q water purification system.

### Preparation of HEWL PNFs and GNPs ink

The HEWL PNFs and GNPs ink were prepared following our previous published procedure.^32^ Typically, HEWL was dissolved in 25 mM HCl (10 mg mL^−1^) followed by filtration through a PES syringe filter (porous size 0.2 μm) to remove undissolved particles. The resulting solution was heated at 80 °C for 72 h with magnetic stirring (1000 rpm) to form HEWL PNFs. The GNPs were prepared *via* a ball milling method. Briefly, 1 mL of 10 mg mL^−1^ PNFs solution was added into a 1.5 mL stainless steel cup with 100 mg of graphite flake sheets, following milling with 30 Hz frequency and 20 balls of 3 mm in diameter in a shaker mill (Mixer Mill MM 400, Retsch, Germany) for 1 h. After milling, the mixture solution was diluted, and the cup was washed by water and transferred to a tube and centrifuged for 60 min at 1 krpm for 3 times to get rid of unexfoliated graphite. The supernatant liquid was collected and then centrifuged at 12 krpm for another 60 min to remove excess PNFs. Finally, the exfoliated GNPs sediment was collected and dispersed to 10 mg mL^−1^ of GNPs, which was used for device fabrication. Note that during the milling step where graphite is exfoliated by PNFs, the PNFs are cut into short fibrils. Also, it is hard to completely remove the dispersing agent from aqueous GNP dispersions and there will thus be residual PNF materials left.

### Device fabrication

Then the conductive carbon paste was printed on clean polyethylene terephthalate (PET) substrates as L-shape pattern which was served as an electrode. Then the PET substrates were treated with plasma for 3 min to increase surface hydrophilicity. Active material dispersion was locally drop casted on the PET films using a tape mask (3 × 1 cm^2^) and dried on a hot plate at 60 °C. The active materials were prepared as follows: 150 μL of a 10 mg mL^−1^ GNP dispersion was mixed with 0 μL, 15 μL, 30 μL, 75 μL of 10 mg mL^−1^ HEWL PNF dispersion, respectively. Similar procedures were used for active layers with the composition of additional salts. 150 μL of a 10 mg mL^−1^ GNP dispersion and 30 μL of a 0.1 M salt solution was mixed prior to drop casting. The corresponding devices are labeled as GNP:NaCl, GNP:CaCl_2_, and GNP:AlCl_3_, respectively. 150 μL of a 10 mg mL^−1^ GNP dispersion, 30 μL of 10 mg mL^−1^ HEWL PNF dispersion, and 30 μL of a 0.1 M salt solution was mixed prior to drop casting. The corresponding devices are labeled as GNP:PNF:NaCl, GNP:PNF:CaCl_2_ and GNP:PNF:AlCl_3_, respectively. The carbon electrodes were extended by metal wires. Finally, all the exposed electrode regions were encapsulated by epoxy glue to avoid direct contact with water.

### Materials characterization

Transmission electron microscopy image was carried out with a FEI Tecnai G2 TF20 UT instrument operated at 200 kV. Scanning electron microscopy (SEM) images were recorded on a Philips XL30 FEG SEM microscope. The film samples were cut to small pieces and adhere by Cu tape to the substrates. All samples were sputter-coated with a thin layer of Pt under argon in a sputter coater (Leica EM SCD 500). X-ray photoelectron spectroscopy (XPS) spectra data were collected on a Kratos Axis Ultra DLD instrument. AFM image was carried out using a Digital Instruments Dimension 3100 atomic force microscope.

### Device characterization

The two-electrode device was put into a 50 mL beaker, then the ultra-pure water was added into the beaker until the water level reached a certain level. The current–voltage, the open-circuit voltage (*V*_oc_) and the short-circuit current (*I*_sc_) characterization of the device was performed with an Autolab Potentiostat with a reference electrode and a counter electrode connection and a working electrode. The working electrode was connected to the upper carbon electrode of the device and the lower carbon electrode of the device was connected to the connected reference electrode and counter electrode. The environmental temperature and relative humidity were about 21 ± 1 °C and 25 ± 5% otherwise mentioned in the text.

## Conflicts of interest

There are no conflicts to declare.

## Supplementary Material

NA-005-D2NA00388K-s001
