# Supplementary material for: Ionovoltaic electricity generation over graphene-nanoplatelets: protein-nanofibril hybrid materials
Source: Nanoscale Adv. 2023 Jan 10;5(3):820–9. doi: 10.1039/d2na00388k (PMC9890733; doi:10.1039/d2na00388k)
Supplement: NA-005-D2NA00388K-s001 [file NA-005-D2NA00388K-s001.pdf]

Electronic Supplementary Material (ESI) for Nanoscale Advances.
This journal is © The Royal Society of Chemistry 2023

Ionovoltaic electricity generation over graphene-nanoplatelet: protein-nanofibril hybrid materials

Lei Wang, Lianlian Liu and Niclas Solin*

Department of Physics, Chemistry, and Biology, Division of Electronic and Photonic Materials, Biomolecular and Organic Electronics, Linköping University, Linköping, 581 83, Sweden

Supporting Information

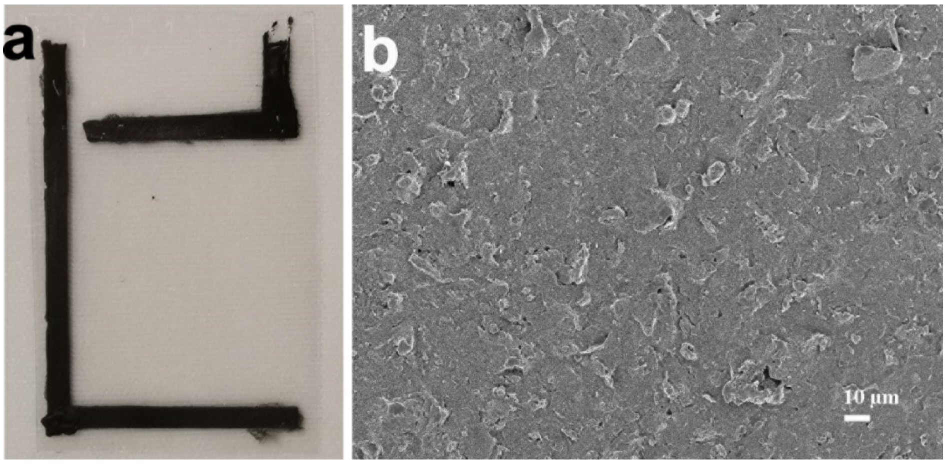

Figure S1. Photo of electrodes on a PET substrate (a). SEM image of surface the of conductive carbon electrode (b).

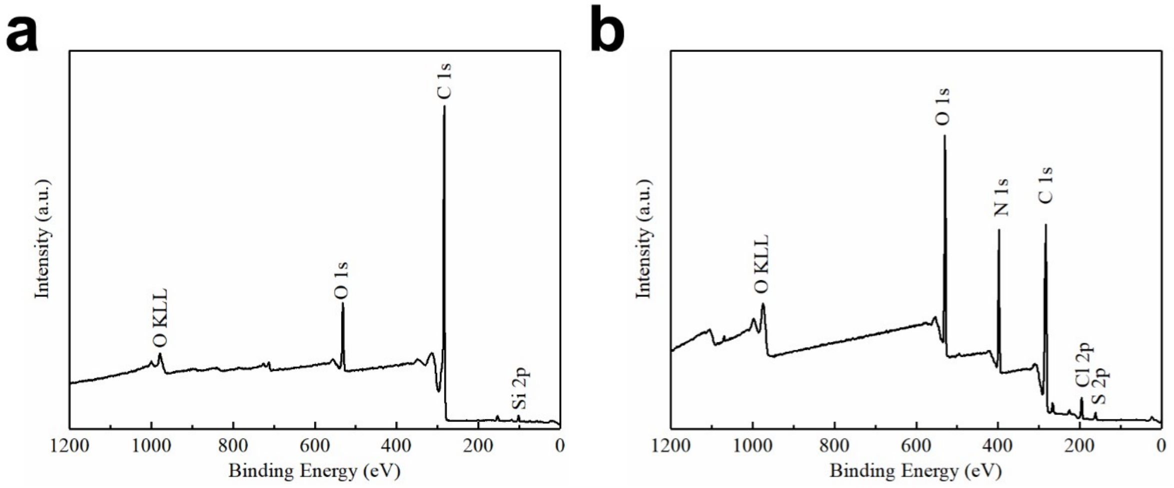

Figure S2 XPS survey spectrum of graphite (a) and PNFs (b) (Si element comes from the Si substrate).

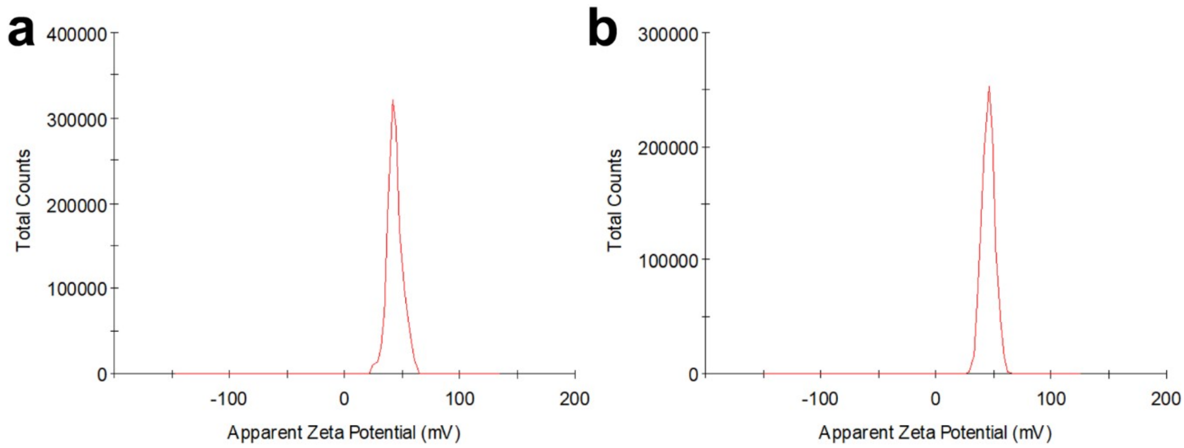

Figure S3 zeta potential distribution curve of the GNP-ink (a) and HEWL PNFs (b) in water dispersion.

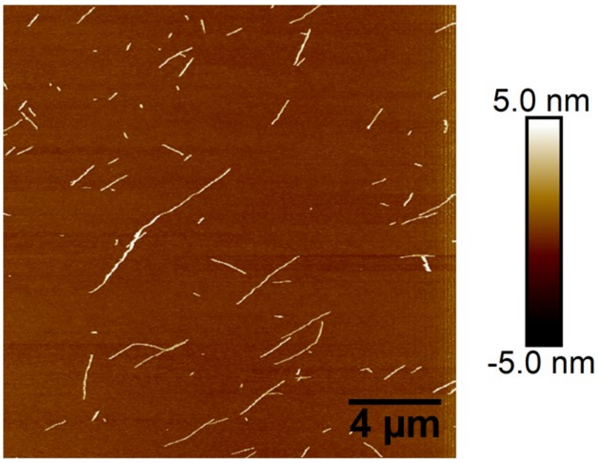

Figure S4 AFM image of HEWL PNFs.

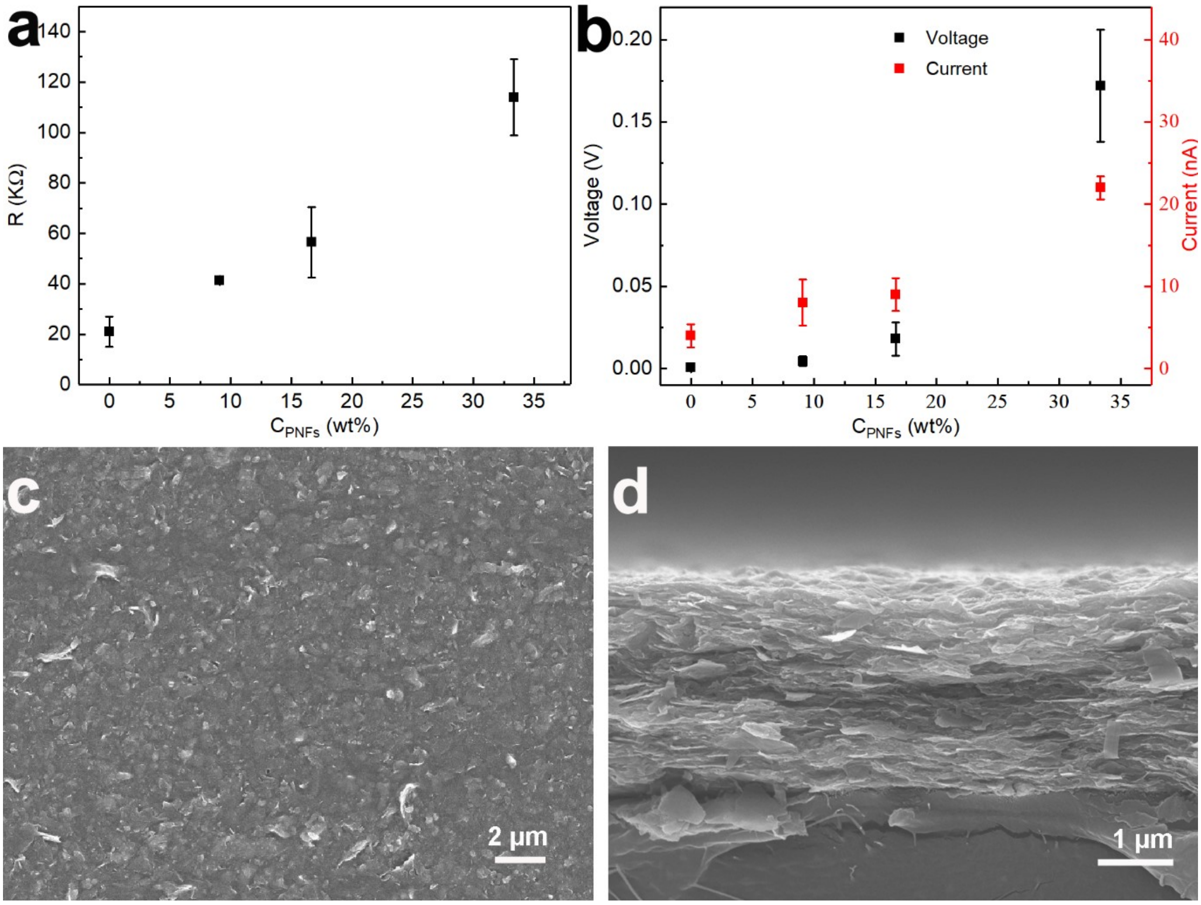

Figure S5 Resistance vs. PNF content (a); V_{oc} and I_{sc} vs. PNF contents (b) (Note that voltage and current curves were obtained sequentially from the same device; first the voltage was measured for 6000 s followed by a measurement of the current for 6000 s. the current plotted in figure S4b is the steady current). SEM images of surface (c) and cross section (d) of a typical GNP:PNF film.

In Figure S5a is shown the effect of addition of PNFs to the original GNP-ink. In the figure the amount of added PNF is indicated; however, it should be noted that the original GNP-ink also contains a small

amount of protein. The resistivity of GNP:PNF films increases with increasing PNF content (figure S5a), which is due to the electrically insulating character of PNFs. The fabricated device is then inset into a 50 mL beaker and about 10 mL of water is injected into the beaker, which covers the bottom part of the device. The V_{oc} and the I_{sc} are then investigated (figure S5b,). Interestingly, when the original GNP-ink is employed as active layer a negligible V_{oc} of 0.5 mV and I_{sc} of 3 nA is obtained. When gradually adding PNFs to the original GNP-ink, thereby increasing the PNF content in the active layer, an increase in both V_{oc} and I_{sc} with increasing PNF content is observed. When the active layer contains up to 16.6wt% of added PNF content, the V_{oc} and I_{sc} increase gradually, reaching an V_{oc} of 0.04 V and a I_{sc} of 10 nA. When increasing the added PNF content up to 33.3wt% the V_{oc} and I_{sc} increases dramatically to 0.17 V and 22 nA. However, in the case of a high PNF loading (33.3wt%) the active layer become unstable in water, and black particles can be observed to be slowly released from the active layer after 1 h keeping in water. So, as the GNP:PNF (with 16.6wt% PNF) offered a good compromise between stability and performance, GNP:PNF with 16.6wt% PNF content is employed as a standard composition and the corresponding device is abbreviated as GNP:PNF device in the latter description.

Figure S5c shows the SEM image of the GNP:PNF (with 16.6 wt% PNF) film surface, where the sheets structure is observed, which reveals the GNPs dispersing on the substrate. Also, a smooth surface is observed, which is due to the PNFs filling in the space between the GNP sheets. Figure S5d show the cross section of GNP:PNF films with sheets structure and thickness about 3 μm.

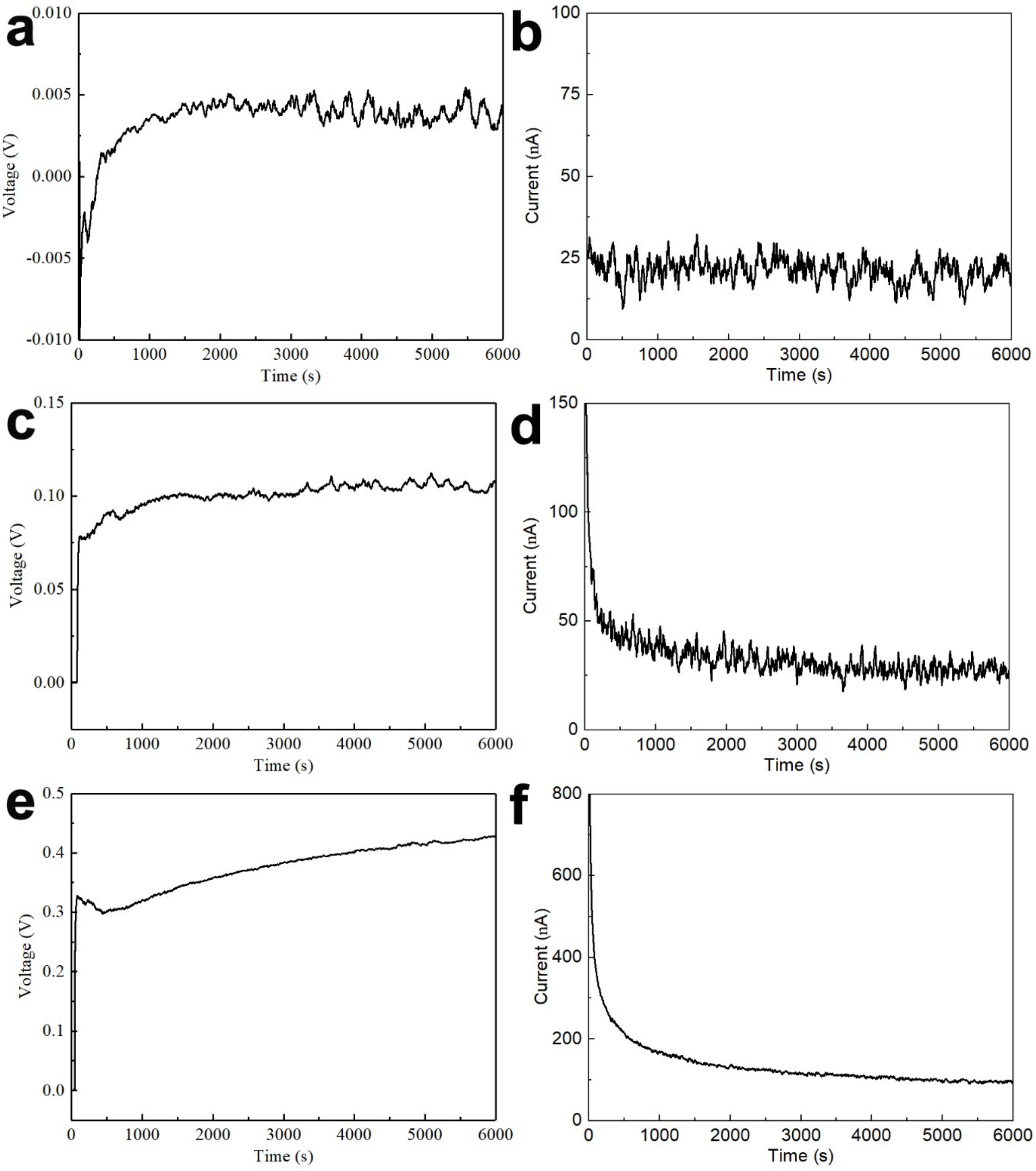

Figure S6 Voltage and current as a function of time for devices with an active layer fabricated from GNP:salt: GNP:NaCl (a, b), GNP:$CaCl_2$ (c, d) and GNP:$AlCl_3$ (e, f). (Note that voltage and current curves were obtained sequentially from the same device; first the voltage was measured for 6000 s followed by a measurement of the current for 6000 s.)

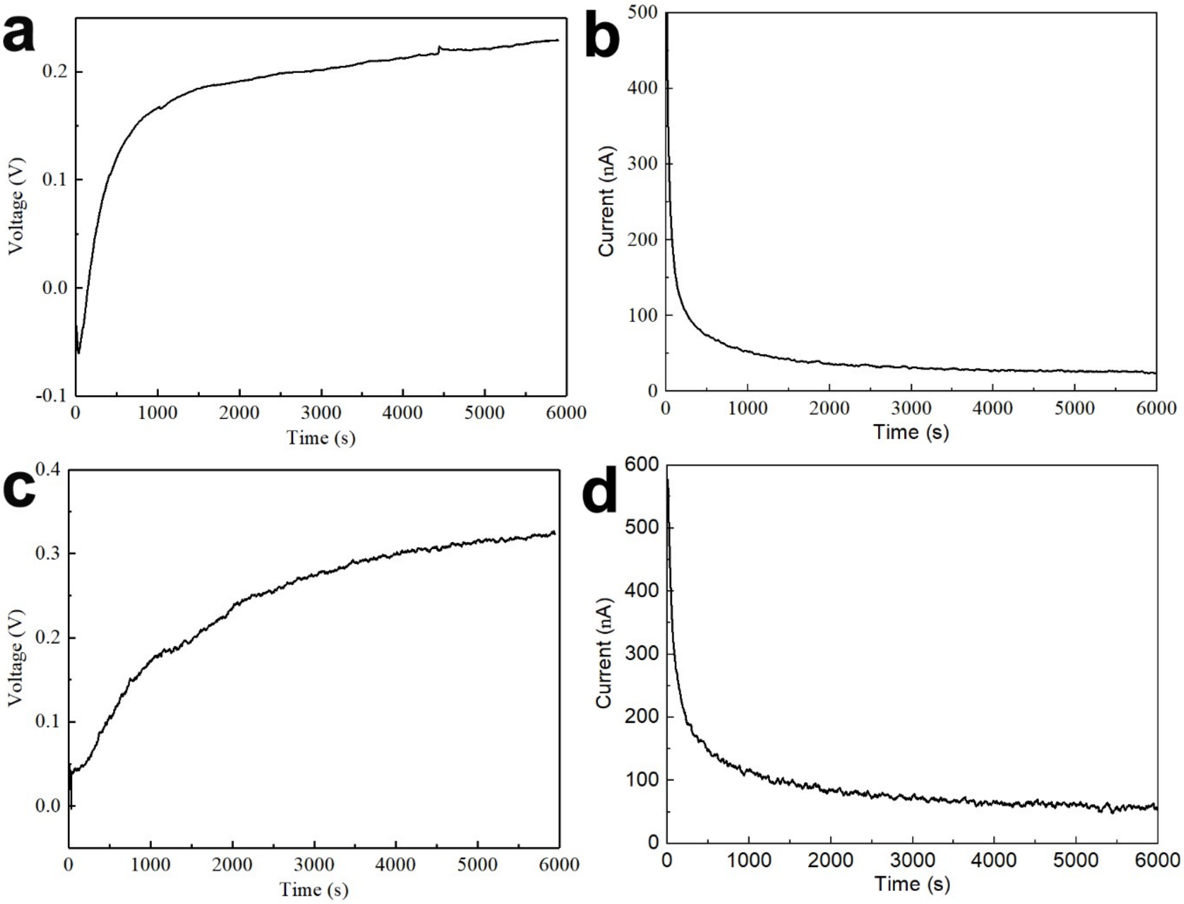

Figure S7 Voltage and current as a function of time for devices with an active layer fabricated from GNP:PNF:salt: GNP:PNF:NaCl (a, b), and GNP:PNF:$CaCl_2$ (c, d). (Note that voltage and current curves were obtained sequentially from the same device; first the voltage was measured for 6000 s followed by a measurement of the current for 6000 s.)

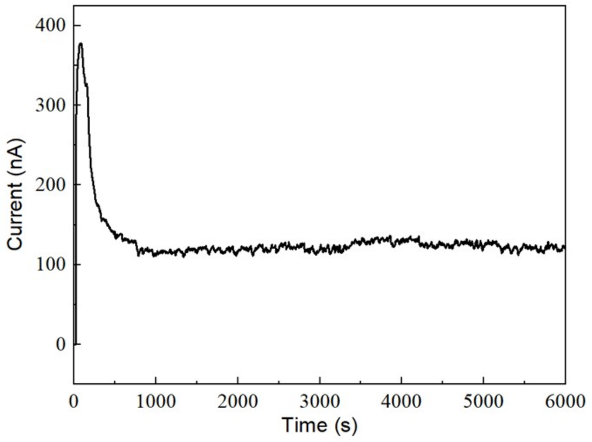

Figure S8 Current as a function of time for GNP:PNF:$AlCl_3$ devices without measuring the V_{oc} first.

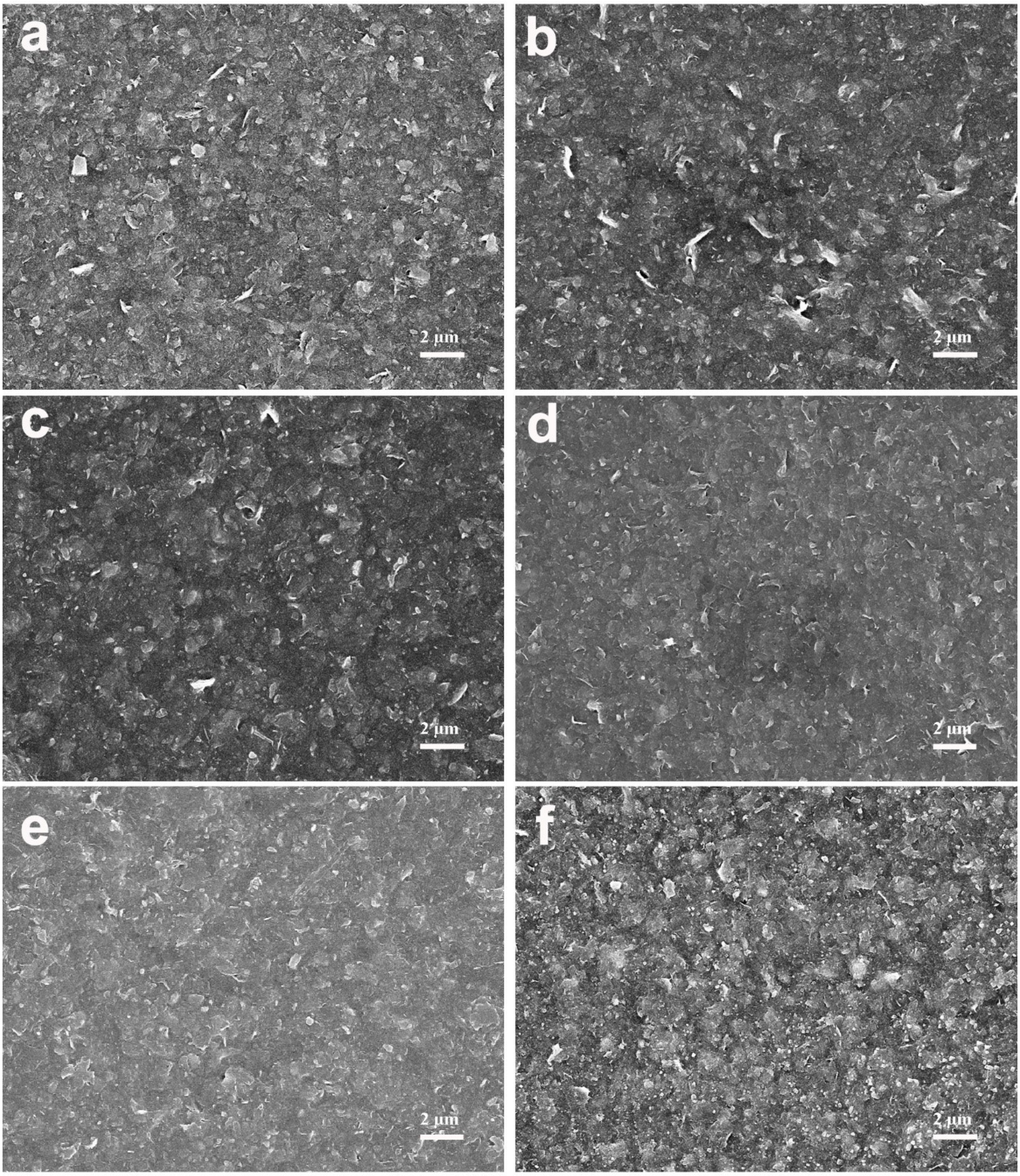

Figure S9 SEM images of films GNP:PNF:$AlCl_3$ (a); GNP:$AlCl_3$ (b); GNP:PNF:$CaCl_2$ (c) ; GNP:$CaCl_2$ (d); GNP:PNF:NaCl (e) and GNP;NaCl (f).

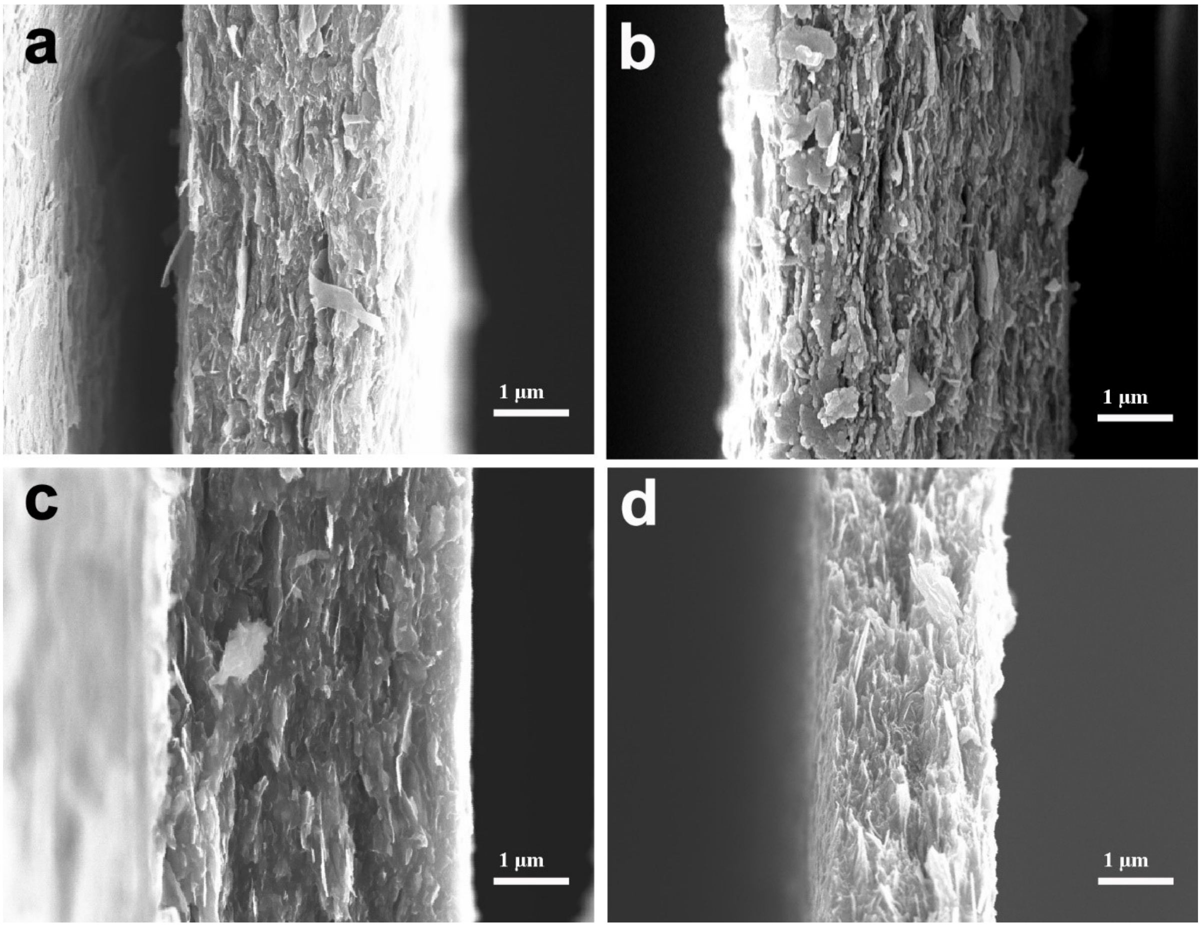

Figure S10 SEM images of films GNP:PNF:$CaCl_2$ (a) ; GNP:$CaCl_2$ (b); GNP:PNF:NaCl (c) and GNP;NaCl (d).

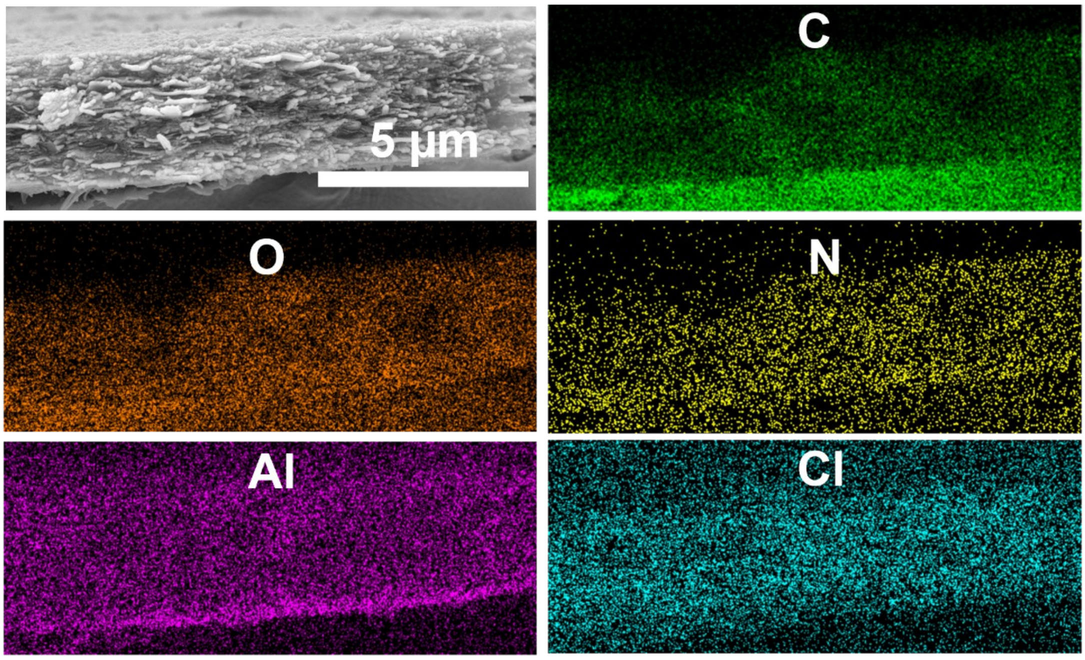

Figure S11. SEM image of cross section of a GNP:$AlCl_3$ film and carbon, oxygen, nitrogen, aluminum and chlorine mapping of the same position.

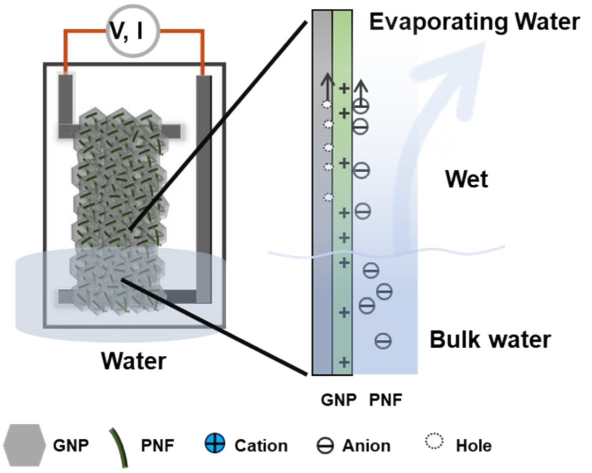

Figure S12, Schematic image of the water evaporation induced electricity generation in device of GNP:PNF.

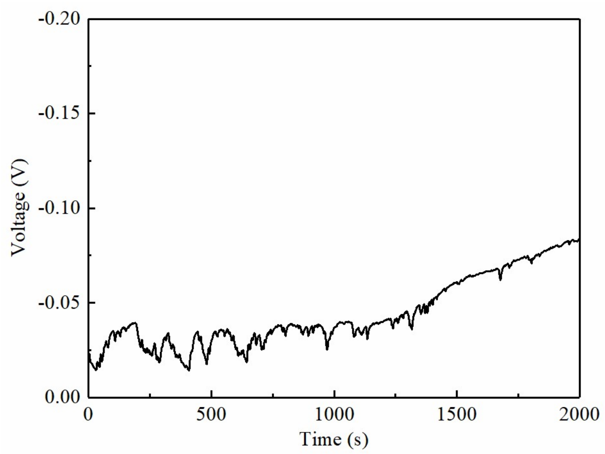

Figure S13. V_{oc} of the GNP:PNF:$AlCl_3$ device as function of time. One end of the device was fix to 40 °C as a hot side and the other end of the device was room temperature (20 °C) as a cold side. The device was exposed to relative humidity of 99%. The induced V_{oc} of the device for thermoelectric effect was about 0.08 V when the temperature difference was 20 °C.

The volage test stops When after exposing the device for more than 2000s, the surface of the active layer saturated with water drops.

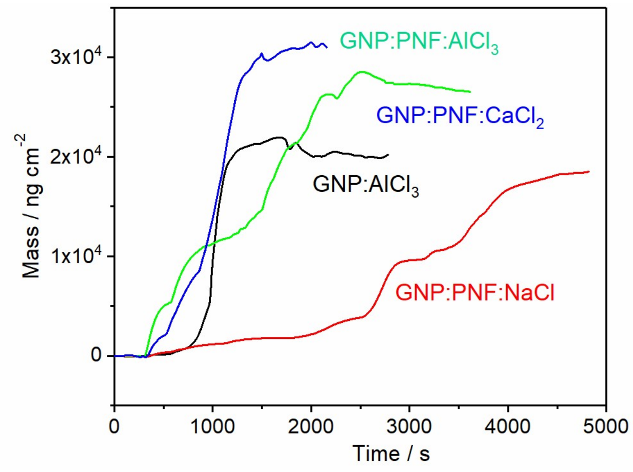

Figure S14 Measured weight change per unit area in different films when the film exposed to relative humidity of 65%.

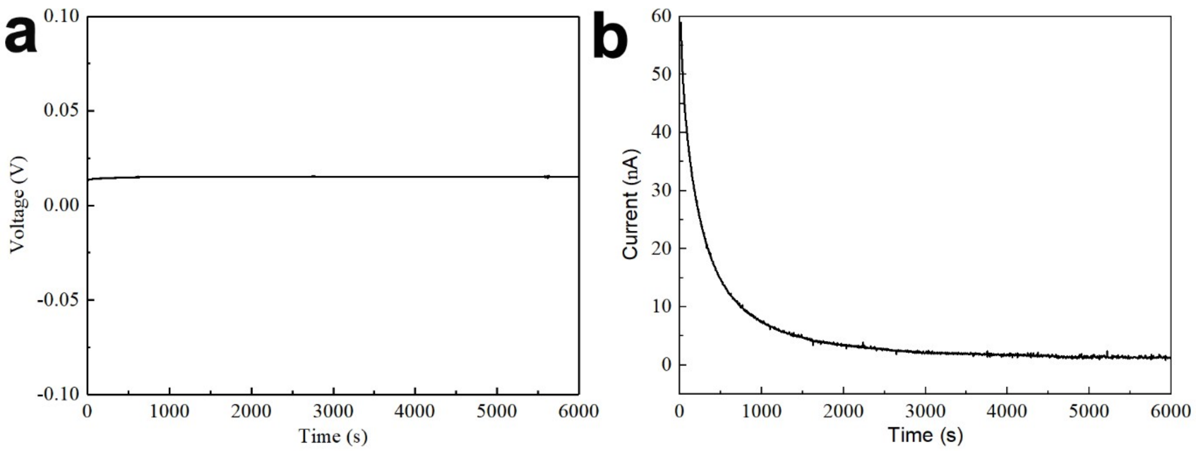

Figure S15 Voltage and current as a function of time for GNP:PNF:$AlCl_3$ devices exposed to relative humidity of 96%.
